# Genomic Imprinting of *Grb10* : Coadaptation or Conflict?

**DOI:** 10.1371/journal.pbio.1001800

**Published:** 2014-02-25

**Authors:** Jon F. Wilkins

**Affiliations:** Ronin Institute, Montclair, New Jersey, United States of America

## Abstract

Mammalian development involves significant interactions between offspring and mother. But is this interaction a carefully coordinated effort by two individuals with a common goal—offspring survival? Or is it an evolutionary battleground (a central idea in our understanding of reproduction). The conflict between parents and offspring extends to an offspring's genes, where paternally inherited genes favor demanding more from the mother, while maternally inherited genes favor restraint. This “intragenomic conflict” (among genes within a genome) is the dominant evolutionary explanation for “genomic imprinting.” But a new study in *PLOS Biology* provides support for a different perspective: that imprinting might facilitate coordination between mother and offspring. According to this “coadaptation theory,” paternally inherited genes might be inactivated because maternally inherited genes are adapted to function harmoniously with the mother. As discussed in this article, the growth effects associated with the imprinted gene *Grb10* are consistent with this idea, but it remains to be seen just how general the pattern is.

## Abstract 2

Knocking out the *Grb10* imprinted gene in a mother compensates for the loss of the same gene in her offspring. Is this evidence of a role for coadaptation in the evolution of imprinting?

## Genomic Imprinting

As diploid organisms, we humans inherit two copies of most of our genes, one from our mother and one from our father. Those two alleles might be the same or different, but in general, it does not matter which allele came from which parent. But, for a small fraction of our genes (∼1% in mammals), it *does* matter which allele came from which parent. These genes are subject to genomic imprinting, an epigenetic phenomenon in which the pattern of expression depends on an allele's parental origin. Genomic imprinting results from differential epigenetic modifications (such as DNA methylation and histone modifications) established separately in the maternal and paternal germ lines during oogenesis and spermatogenesis, respectively. After fertilization, the genome undergoes large-scale epigenetic reprogramming; the differences that survive can be propagated across cell divisions throughout development. The result is that the genome in each cell contains certain loci where the two alleles are in different epigenetic states, and therefore interact differently with the transcriptional and regulatory machinery.

In the simplest cases, one imprinted allele is expressed, while the other is silent, but many imprinted loci exhibit more complex expression patterns. Some, like the *Gnas* locus [1], encode multiple gene products, including splice variants transcribed from different promoters, each with a different pattern of imprinting. At others, silencing of a protein-coding transcript results in *cis* from the production of an anti-sense, non-coding RNA transcript, which may be processed into various small RNA products, such as microRNAs and snoRNAs (e.g., the *Ube3a-ATS* transcript, which regulates genes associated with Angelman and Prader-Willi Syndromes [2]). Some loci show tissue-specific imprinting, with monoallelic expression in some cell types, and biallelic expression in others. And, of course, some imprinted loci combine all of the above.

## More, and More Varied, Imprinted Genes

Over the past two decades, more than 100 imprinted genes have been identified in mice, and over 50 in humans. Many imprinted genes affect early growth and development in ways that are consistent with the predictions of the Kinship Theory of Imprinting (see Box 1), but it is increasingly clear that imprinted genes have systematic effects on other phenotypes. For example, imprinted genes affect various aspects of metabolism [3], extending into adulthood, and many imprinted genes are expressed in the central nervous system (CNS), with major effects on cognition and behavior [4].

**Box 1. The Kinship/Conflict Theory of Imprinting**

The Kinship (or Conflict) Theory of Imprinting [26],[27] was first applied to fetal/placental genes that play an active role in soliciting resources from the mother during pregnancy [28]–[30]. In mammals, the optimal demand on maternal resources is different for maternally and paternally inherited alleles in an offspring: natural selection favors alleles that demand more maternal resources when paternally inherited, and fewer resources when maternally inherited. Maternally inherited alleles favor limited demand because each of the mother's other offspring has a 50% chance of carrying an identical copy of the allele. Paternally inherited alleles favor slightly greater demand, since some of the mother's other offspring could have different fathers, so their chance of carrying an identical copy of the paternally inherited allele is less than 50%.

At unimprinted loci, natural selection drives demand to a point between the optima for maternally and paternally inherited alleles. However, genomic imprinting allows alleles to evolve two separate expression patterns. For loci where the gene product acts as a fetal growth enhancer, the evolutionarily stable pattern is monoallelic expression from the paternally inherited allele. At a growth-suppressing locus, it is expression from the maternally inherited allele [31]–[34].

**Figure 1:**
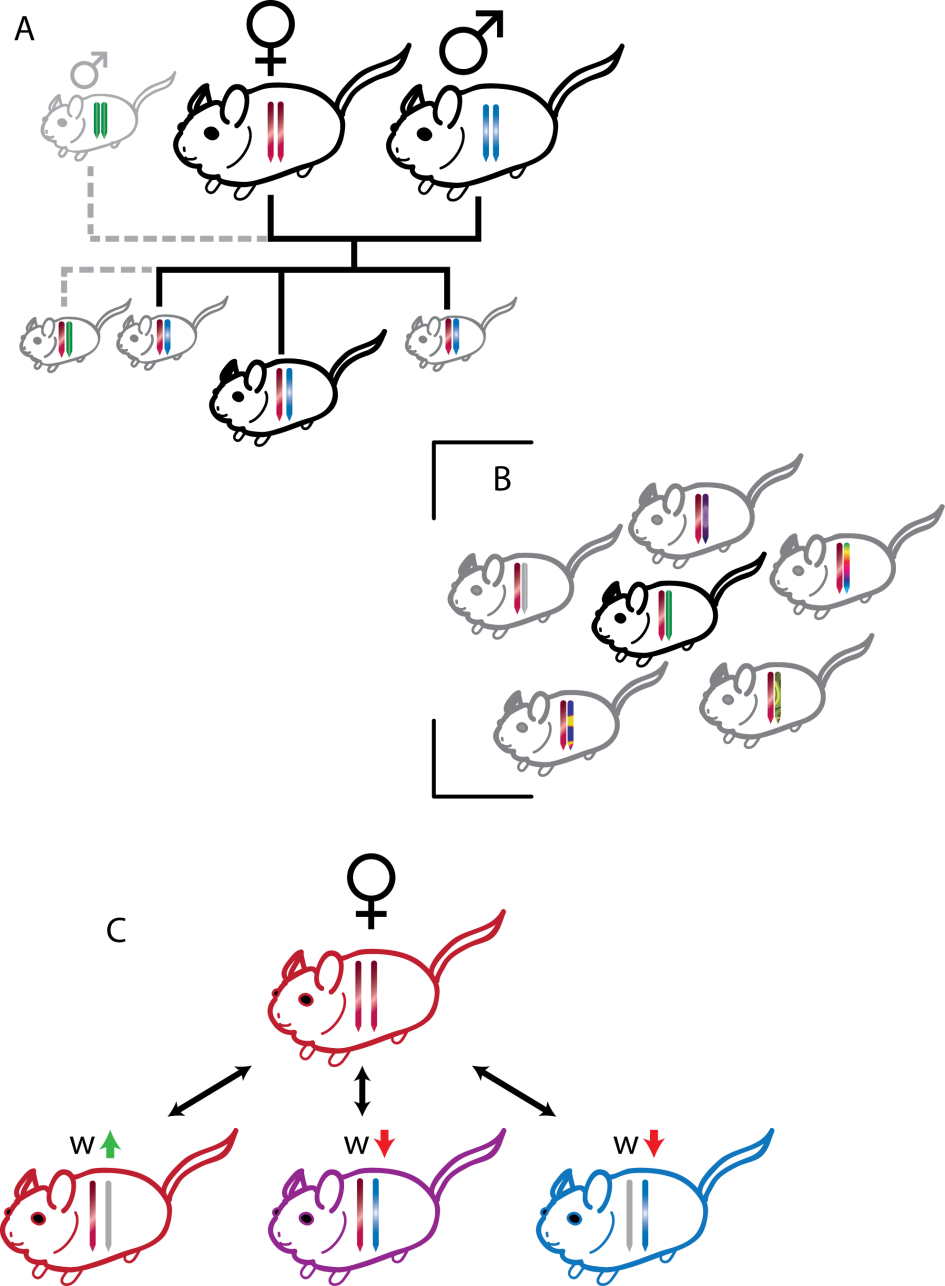
Illustrations of three scenarios that could favor the evolution of imprinted gene expression. The genome of each individual is represented by two symbolic chromosomes, with the left chromosome representing maternally inherited alleles, and the right chromosome paternally inherited alleles. Different colors and patterns on the chromosomes are used to suggest different allelic variants. (A) The Kinship Theory applied to fetal growth modifiers. The focal offspring is more closely related to its litter-mates through its maternally inherited allele than through its paternally inherited allele. Paternally inherited alleles favor greater demand on maternal resources, because their inclusive fitness is less affected by the indirect costs of reducing the pool of maternal resources available to the mother's other offspring. (B) One way the Kinship Theory might explain certain imprinted gene effects in adults. If demographic processes (e.g., sex-biased migration) create groups that are more closely related through their maternally than paternally inherited alleles, imprinted expression could be favored at genes that affect the fitness of other group members (e.g., by favoring “cooperation”). (C) The central idea of the Coadaptation Theory. The mother, who carries two “red” alleles has three hypothetical offspring, each of which inherits one “red” allele from her, and one “blue” allele from the father. The offspring on the left silences its paternally inherited (blue) allele, and thus expresses the “red” phenotype, matching the mother. The offspring at the center and right show biallelic (red+blue = purple) and paternal (blue) expression, respectively, resulting in phenotypes that do not match that of the mother. Paternal silencing is favored if phenotype matching (or complementarity) leads to increased fitness.

Some of these phenotypes, such as imprinted gene effects on suckling and weaning behaviors, can be understood through straightforward extensions of the Kinship Theory, with the intragenomic conflict over maternal resource demand continuing after birth (Figure 1A) [5],[6]. But what about other phenotypes, such as effects on adult behavior?

In principle, the logic of the Kinship Theory applies to any trait that affects the fitness of other, related individuals. In fact, in its general form, the theory is simply an extension of standard inclusive-fitness theory [7],[8], where inclusive fitness effects are calculated separately for maternally and paternally inherited alleles [9]. This generalization of the Kinship Theory makes sense once we recognize that most relatives (excepting full siblings and descendants) are related either matrilineally or patrilineally—for example, a cousin on my mother's side is a pretty close relative to my maternally inherited alleles, but a complete stranger to my paternally inherited alleles.

In practice, however, the application of the Kinship Theory to non-growth-related traits is challenging. The models quickly become complicated, and it is not always obvious how to connect them to empirical data. Specific biological predictions can vary, depending on factors such as sex-biased dispersal in structured populations and the scale of (geographically or socially) local competition for resources [10]–[13]. For example, imagine a species where males disperse over long distances every generation. At the local scale, we would find that individuals were more closely related through their maternally inherited alleles than their paternally inherited ones (Figure 1B). We would expect the elaboration of traits involved in cooperation and resource sharing to be preferentially favored by those maternally inherited alleles. Of course, real systems will typically be more complicated, and will not lead to this sort of robust, qualitative prediction. Even seemingly benign factors—such as whether the population has overlapping or non-overlapping generations—can substantially alter conclusions of the models [14]. The testing of these models' predictions requires quantitative measurements of relatedness, social interaction, reproduction, and resource allocation, which will be challenging in most natural populations [15].

Thus, in addition to continuing to examine the implications of the Kinship Theory, it is important to explore alternative hypotheses that might complement, or even supersede, the Kinship Theory within specific contexts. So far, most of the alternative hypotheses proposed have not proven to have predictive or explanatory power that would justify replacing or augmenting the Kinship Theory [16]. However, one notable exception is the Coadaptation Theory [17], which in their recent *PLOS Biology* article, Cowley and colleagues set out to test [18].

## Mother-Offspring Coadaptation

The basic idea behind the Coadaptation Theory is that the mother-offspring system, when taken as a whole, functions better when the components of the system are aligned with each other. In terms of imprinting, this means “allele matching,” driven by the close interaction between mother and offspring during early development. For example, imagine a gene expressed in both mother and offspring, where the gene products interact (perhaps only indirectly, through effects on a shared phenotype). If there is substantial functional variation among alleles in the population, we might expect certain combinations of alleles to be more compatible than others. We might also expect the functions of the same allele, expressed in mother and offspring, to be more compatible, since their compatibility is constantly subjected to selection. Different alleles, which co-occur less frequently, might accumulate incompatibilities. Silencing the paternally inherited copy in the offspring would avoid interaction between that allele and a potentially incompatible allele in the mother (Figure 1C). It is like avoiding conflict at holiday gatherings by letting your spouse do most of the talking when you visit the in-laws.

Theoretical models have shown that this type of system can, indeed, favor imprinted gene expression, at least under certain conditions [17]. The question then becomes, how often do those conditions hold? Are there imprinted genes for which natural selection for coadaptation was likely a more significant factor than differential inclusive fitness effects on maternally and paternally inherited alleles?

## Compensatory Pleiotropy

The work by Cowley and colleagues [18] examines the effect of a loss-of-function mutation of the imprinted *Grb10* gene in mice. *Grb10* is pleiotropic, with at least three distinct phenotypic effects. It is expressed exclusively from the maternally inherited allele in fetal and extra-embryonic tissues during pregnancy, where it restricts growth, consistent with predictions from the Kinship Theory [19],[20]. It is also maternally expressed in the peripheral tissues of adults, where it plays a role in glucose homeostasis and insulin signaling [21]. Of particular interest here is the expression of *Grb10* in the mammary epithelium of lactating females [18]. Adult mice also express *Grb10* in their CNS, specifically from the paternally inherited allele [22], where it affects adult behavior [23].

This study focuses on the growth effects of the maternally expressed transcripts in mother and offspring, using a well-characterized genetic construct to knock out *Grb10*. The authors implemented various cross-fostering arrangements to separate two major effects of the gene product: (1) expression of *Grb10* in offspring suppresses demand for maternal resources; and (2) expression of *Grb10* in the mother's mammary epithelium enhances the maternal nutrient supply during lactation.

As expected, knocking out *Grb10* in offspring results in mice that are larger than wild type. Knocking it out in the mother (but not the offspring) produces small mice. The exciting result is that when you combine the two knockouts—eliminating maternally inherited *Grb10* in both mother and offspring—the two effects cancel each other out, and the offspring recover their wild-type body size. These equal-and-opposite effects of the two knockouts suggest the type of compensatory pleiotropy described by the Coadaptation Theory, but it is still an open question whether such compensation is typical of natural allelic variation at the locus.

For example, interpolating between the wild-type and knockout results, we might assume that the growth enhancement resulting from a 25% reduction in *Grb10* expression in the offspring would be offset by the growth restriction resulting from a 25% reduction in *Grb10* expression in the mother. The question then becomes, what is the relationship between the regulatory elements responsible for dosage in these two conditions? Will a given mutation typically have similar effects on expression or activity in the two tissues? If so, the case for coadaptation is compelling. On the other hand, if the two activities are largely independent (e.g., if expression of *Grb10* in mother and offspring is controlled by two completely different sets of *cis*-acting enhancer elements), then the phenotype of the double knockout will seem more like an interesting coincidence.

## Two Types of “Evolution of Imprinting”

The striking data presented by Cowley and colleagues are consistent with a role for coadaptation in the evolution of imprinting, and their results will hopefully prompt more research in this area. So what future results would lead us to conclude that coadaptation has played a major role in the evolution of imprinting at the *Grb10* locus or was a major factor in the evolution of imprinting in general?

The “evolution of imprinting” actually refers to two distinct processes. The first is the acquisition of imprinted gene expression at a locus—the evolutionary transition from being unimprinted to imprinted. The second is the evolution at a locus after it has become imprinted. Coadaptation could potentially play an important role in either of these processes.

If coadaptation drove the acquisition of imprinting at *Grb10*, this implies that the complementary phenotypic effects of *Grb10* expression in mother and offspring predated the evolution of imprinted gene expression. In this scenario, genomic imprinting evolves because, by increasing the allelic match between mother and offspring, it enhances this complementarity. Because *Grb10* would already have its growth-suppressing effect in the offspring, it would be reasonable to say that Kinship and Coadaptation both contributed to the selective pressure favoring paternal silencing at the locus.

An alternative scenario follows the “Growth First” theory of imprinting [24], where paternal silencing of *Grb10* is driven by its growth-suppressing function in the offspring. Maternal expression in the peripheral tissues of adults is, at first, an epiphenomenon not requiring an adaptive (selective) explanation. Then, variation among *Grb10* alleles creates selection to canalize, or buffer, the resulting variation in growth rates. This selects for *Grb10* to acquire its novel, pleiotropic function in the mother, where it enhances resource provisioning.

Distinguishing between these scenarios requires determining the order in which *Grb10* acquired these two features—imprinting and coadaptation. One possibility involves a comparative, taxonomic approach. Under the first scenario, we might find species where *Grb10* exhibits complementary growth effects in mothers and offspring, but where the locus is not imprinted. Under the second, we might find species where *Grb10* is imprinted, but not expressed in mammary tissues. A recent study found that *Grb10* is widely expressed in the tissues of the Tammar wallaby, a marsupial, where its expression appears not to be imprinted [25], but its function in this species remains unknown.

It is important to keep in mind that evolutionary explanations are rarely mutually exclusive. Evolutionary Biology is a historical science, and identifying “the cause” of a unique evolutionary event, such as the acquisition of imprinting at *Grb10*, is analogous to trying to identify “the cause” of the French Revolution. Each unique event involves multiple selective factors, as well as a healthy dose of chance.

The power of the Kinship Theory is that it makes at least some sense of many of the large-scale patterns associated with imprinting, across disparate genes and species, including analogous phenomena in plants and insects. The work by Cowley and colleagues in *PLOS Biology* [5] represents the first real attempt at testing the Coadaptation Theory. As this perspective is brought to bear on other cases, we will see if it is able to bring additional order to the zoo of imprinted genes and their phenotypes.
